# Seasonality of human sleep: Polysomnographic data of a neuropsychiatric sleep clinic

**DOI:** 10.3389/fnins.2023.1105233

**Published:** 2023-02-17

**Authors:** Aileen Seidler, Katy Sarah Weihrich, Frederik Bes, Jan de Zeeuw, Dieter Kunz

**Affiliations:** ^1^Sleep Research and Clinical Chronobiology, Berlin Institute of Health, Institute of Physiology, Charité – Universitätsmedizin Berlin, Corporate Member of Freie Universität Berlin, Humboldt-Universität zu Berlin, Berlin, Germany; ^2^Clinic for Sleep & Chronomedicine, St. Hedwig Hospital, Berlin, Germany

**Keywords:** sleep, REM, polysomnography, season, human, circadian, slow wave sleep, clinical data

## Abstract

While short-term effects of artificial light on human sleep are increasingly being studied, reports on long-term effects induced by season are scarce. Assessments of subjective sleep length over the year suggest a substantially longer sleep period during winter. Our retrospective study aimed to investigate seasonal variation in objective sleep measures in a cohort of patients living in an urban environment. In 2019, three-night polysomnography was performed on 292 patients with neuropsychiatric sleep disturbances. Measures of the diagnostic second nights were averaged per month and analyzed over the year. Patients were advised to sleep “as usual” including timing, except alarm clocks were not allowed. Exclusion criteria: administration of psychotropic agents known to influence sleep (*N* = 96), REM-sleep latency > 120 min (*N* = 5), technical failure (*N* = 3). Included were 188 patients: [46.6 ± 15.9 years (mean ± SD); range 17–81 years; 52% female]; most common sleep-related diagnoses: insomnia (*N* = 108), depression (*N* = 59) and sleep-related breathing disorders (*N* = 52). Analyses showed: 1. total sleep time (TST) longer during winter than summer (up to 60 min; not significant); 2. REM-sleep latency shorter during autumn than spring (about 25 min, *p* = 0.010); 3. REM-sleep longer during winter than spring (about 30 min, *p* = 0.009, 5% of TST, *p* = 0.011); 4. slow-wave-sleep stable winter to summer (about 60–70 min) with 30–50 min shorter during autumn (only significant as % of TST, 10% decrease, *p* = 0.017). Data suggest seasonal variation in sleep architecture even when living in an urban environment in patients with disturbed sleep. If replicated in a healthy population, this would provide first evidence for a need to adjust sleep habits to season.

## 1. Introduction

Various physiological processes and medical conditions in humans are known to exhibit seasonal patterns. This also includes medical conditions that affect sleep. Insomnia is a highly prevalent disturbance in the general population (Schlack et al., 2013) but results on its seasonal pattern are unclear. On the one hand, a study comparing weekly self-reported measures between January and August in Norway (69°N latitude) found seasonal differences in insomnia (Friborg et al., 2012), whereas, on the other hand, a large (2,614 participants) epidemiological study in Japan (36°N) did not find an association between insomnia and seasonality (Itani et al., 2016). Another common sleep-related disorder affected by seasonality is obstructive sleep apnea, reported to increase in winter and decrease in summer (Cassol et al., 2012). Naturally, seasonality is evident in seasonal affective disorder (SAD) including lengthened sleep and weight gain during winter (Rosenthal et al., 1984), which is more prevalent in people living at higher latitudes (Rosen et al., 1990). SAD patients experience rapid positive effects of light therapy (Rosenthal et al., 1984), with effects being more pronounced using light of high intensity, long exposure early in the day, and exposure to the eyes rather than the skin (Wehr and Rosenthal, 1989).

Light exposure is known to impact sleep architecture (Dijk et al., 2012; Chellappa et al., 2013; Wams et al., 2017). In healthy humans, the influence of the length of scotoperiods (periods of darkness) and melatonin secretion was already demonstrated in laboratory settings. Participants’ sleep period time (SPT) became 3.3 h longer under 10 h photoperiods (period of light) than under 16 h photoperiods (Wehr, 1991). The most important factor for changing sleep durations, apart from sleep debt, might therefore be naturally changing light exposure throughout the year. The length of photoperiods and scotoperiods inversely vary over the year due to the skewed rotation axis of the earth relative to its plane of orbit around the sun. Light plays a significant role in adjusting to seasonal changes since light input to the retina is the main zeitgeber (time cue) for the biological clock. Circadian entrainment of the biological clock is mediated by the suprachiasmatic nucleus (SCN), which controls the secretion of melatonin by the pineal gland (Czeisler, 1995; Maywood et al., 2021). Melatonin is then suspected to synchronize the clocks in the cells of the periphery (Lewy et al., 1992). The hormone is secreted exclusively during scotoperiods (Wehr, 1991) and was shown to vary across the seasons in peak concentration (Adamsson et al., 2017) and duration of secretion (Kauppila et al., 1987). In addition, the SCN itself has been shown to vary with seasons in its volume and in the number of vasopressin-immunoreactive neurons it contains (Hofman and Swaab, 1992).

While biological processes and findings in laboratory settings would speak for seasonal changes in human sleep duration throughout the year, the expression of such changes in real life and especially in urban settings is contested. In the 1990s no difference in sleep timing and length was found when controlling for the midpoint of the nocturnal period (Wehr et al., 1995). A substantial amount of work had shown inconsistent results on seasonal variation of melatonin levels over the year for humans living in an urban environment (Wehr, 1997). Thus, the function of melatonin on human seasonality was questioned in general. The recently reported association of subjective seasonality and subjective sleep duration with individual melatonin levels determined by the degree of pineal calcification (Kunz et al., 2021) may have provided the missing link. In that study, the subjective sleep duration of healthy adults varied by approximately 1 h over the year. The next step is to investigate seasonal variations in objectively measured sleep parameters.

Investigating the sleep architecture of large patient groups, while not transferable to assessments of seasonality in a healthy population, would allow us to obtain some preliminary observations. Previously, variations were found between seasons in rapid eye movement (REM) sleep duration and REM latency for men with erectile problems and some with suspected sleep apnea in the subtropical climate of Israel (Askenasy and Goldstein, 1995). REM duration was shorter in spring and summer and longer in autumn and winter. REM latency, on the other hand, was longer in autumn and winter. However, these findings were not replicated in a group of 706 unselected apnea patients (Herer and Lavie, 1997). Both studies examined transient variations by looking at changes in sleep parameters grouped by season. Of special interest are REM sleep parameters, since REM sleep is known to be mainly under circadian regulation (Endo et al., 1981; Dijk and Czeisler, 1995; Bes et al., 1996; Khalsa et al., 2002), being entrained by the daily light-dark cycle.

Our study aimed to determine objective sleep duration and architecture over 1 year in a large group of patients attending a neurologic/psychiatric sleep laboratory. In comparison to other similar studies, patients’ usual environment was not manipulated before the polysomnography (PSG) recording and PSG data were analyzed per month.

## 2. Methods

### 2.1. Patients

In 2019 a standard diagnostic three-night PSG was performed on a total of 292 patients in the sleep laboratory of the Clinic for Sleep & Chronomedicine at the St. Hedwig Hospital in Berlin [52°N, 13°E, shortest and longest photoperiod of 2019 (Thorsen, 2022): 7:39:08 h (22nd Dec) resp. 16:50:00 h (21st Jun)]. All included patients had given their written and informed consent to analyze and publish their data.

Exclusion criteria were: intake of drugs known to disturb sleep, especially REM sleep (*N* = 96: 64 with antidepressants, 13 with dopamine agonists, 4 with neuroleptics, 4 with anticholinergic medicine, 2 with stimulants, and 9 with other medications); technical failure (*N* = 3), and REM latency longer than 120 min (*N* = 5). The latter was excluded as a possible indicator of skipped REM (Dement and Kleitman, 1957; Roffwarg et al., 1966). Included were 188 patients: 98 women and 90 men, ages 17–81 years (mean ± standard deviation, *M* ± *SD* = 46.6 ± 15.9 years). Table 1 shows the demographics of patients including the number of patients by month, distribution of gender and age, sleep-related diagnoses, and pharmaceuticals taken during the PSG.

**TABLE 1:** Demographic information about the patients included in the study for 2019.

	Demographics by month [N (%)]							Medication during PSG	
Month	Total	Female	Male	<60 years	≥60 years	Diagnoses	*N*	Medication	*N*
January	20	12 (60.0)	8 (40.0)	13 (65.0)	7 (35.0)	Insomnia	108	No Medication	92
February	13	3 (23.1)	10 (76.9)	11 (84.6)	2 (15.4)	Depression	59	Antihypertensive Drugs	27
March	13	8 (61.5)	5 (38.5)	7 (53.8)	6 (46.2)	Sleep-Related Breathing Disorders	52	Beta Blockers	16
April	15	9 (60.0)	6 (40.0)	11 (73.3)	4 (26.6)	Restless Legs Syndrome		Thyroid Medication	18
May	12	7 (58.3)	5 (41.7)	11 (91.6)	1 (08.3)		20	Melatonin	9
June	18	8 (44.4)	10 (55.6)	14 (77.8)	4 (22.2)	Periodic Limb Movement Disorder	36	Other Medication	56
July	19	8 (42.1)	11 (57.9)	14 (73.7)	5 (26.3)				
August	13	8 (61.5)	5 (38.5)	9 (69.2)	4 (30.8)	REM Sleep Behavior Disorder	19		
September	12	5 (41.7)	7 (58.3)	11 (91.7)	1 (08.3)				
October	15	6 (40.0)	9 (60.0)	11 (73.3)	4 (26.7)		15		
November	18	13 (72.2)	5 (27.8)	15 (83.3)	3 (16.7)	Other			
December	20	11 (55.0)	9 (45.0)	19 (95.0)	1 (05.0)				
**2019**	**188**	**98 (52.1)**	**90 (47.9)**	**146 (77.7)**	**42 (22.3)**				

Patients may have had multiple sleep-related diagnoses and may have taken multiple types of medications.

Number of patients (in total numbers (N) and percent per month (%)) for each month and the distribution of gender (self-reported female or male) and age group (under 60 years old and over or 60 years old at the time of the polysomnography). No significant difference of gender and age group between months (*p* > 0.05).

Sleep-related diagnoses: Number of patients diagnosed with sleep-related diseases. Insomnia is predominant as it is the most common reason for patients to be referred to the clinic, followed by depression and breathing disorders.

Medications: Total number of patients taking medication types during the polysomnography (PSG) night used for the study. Bold values represent the total number of patients participating in the study.

The reported diagnoses were obtained from the medical reports provided to the patients at the end of their diagnostic stay in the clinic. Diagnoses resulted from questionnaires filled out before admittance to the clinic, provided medical documents, anamnesis by the doctor, and analysis of the second PSG night. The distribution of most diagnoses did not show noticeable patterns throughout the year. The exception was insomnia, with less patients toward the start of the year and more patients toward the end of the year (*N_*MeanByMonths*_* = 9; *N*_*Feb*_ = 5, *N_*Jun*_* = 9, *N*_*Nov*_ = 13; *N*_*Spring*_ = 21, *N*_*summer*_ = 31, *N*_*autumn*_ = 35, *N*_*winter*_ = 21).

### 2.2. Polysomnography and sleep parameters

Polysomnography recordings were conducted using a Rembrandt system (Monet 24-CPU hardware, TMS International, Enschede, Netherlands and Rembrandt 7.5 software, Medcare Automation, Amsterdam, Netherlands). Patients were measured three nights in a row. First nights in the laboratory serve as an adaptation to the artificial lab situation and third nights are usually used for the evaluation of newly introduced treatment procedures or medications. Second nights are used for diagnosis and were used for analysis in this study. Due to technical issues in the second night, the third night was used in 16 patients.

As a neuropsychiatric sleep lab, we focus on the representative sleep of our patients. Thus, our standard protocol encourages patients to sleep during their preferred time and to sleep in. After the EEG-setup, “lights-out” is performed at a time individually determined by the preference of the patient. Alarm clocks were not allowed, however, some patients insisted to be woken up at their request. This behavior was discouraged by informing patients that a full night’s sleep is important for diagnostic reasons, but as a compromise, the latest possible wake-up time was agreed upon. Taking the patient’s preferred sleep times during weekends and holidays as a baseline, the protocol allows for the assessment of the patient’s “usual” sleep ability, timing, and length. The rooms in the sleep laboratory have heaters and patients have access to the regulation but do not have air conditioning. Windows (facing east or west) can be fully blinded or opened.

Data were visually scored using the guidelines of the American Academy for Sleep Medicine Version 2.5 (Iber et al., 2007). Standard sleep parameters are listed in Table 2. Sleep Period Time (SPT) is defined as the time between sleep onset (the first two consecutive min of N2, N3, or REM) and the end of sleep (the last two consecutive min of N1, N2, N3, or REM). Total sleep time (TST) encompasses the summed duration of N1, N2, N3, or REM between lights off and lights on. The time between the lights being turned off and sleep onset is defined as sleep latency and the time between sleep onset and the first REM episode as REM latency. Sleep efficiency is defined as the percentage of TST within SPT. REM stability was calculated by dividing the number of REM to not-REM transitions (thus, including transitions from REM to NREM and wake episodes) by REM in minutes. N3 is indicated as slow wave sleep (SWS). The sleep stages were analyzed both as their total duration in minutes (min) and as their percentage of TST (%). Arousal index counts the number of EEG-arousals per hour and the awakening index is the number of transitions from a sleep stage to a wake-episode per hour. For the sleep parameter of the periodic limb movement (PLM) index (number of PLM per hour), only data points of patients with PLM-Disorder (whose PLM index ≥ 5 n/h) were kept.

**TABLE 2:** Monthly means and results of the linear mixed model for each sleep parameter.

Sleep parameter (mean ± SD)	Jan	Feb	Mar	Apr	May	Jun	Jul	Aug	Sep	Oct	Nov	Dec	2019	Linear mixed model
TST (min)	430.9 ± 61.2	408.0 ± 43.4	426.3 ± 82.9	376.4 ± 102.1	413.8 ± 69.6	369.2 ± 60.0	412.8 ± 87.8	398.8 ± 75.7	407.6 ± 83.8	409.9 ± 54.6	408.9 ± 65.3	430.4 ± 71.2	408.3 ± 73.2	*F* (11,176) = 1.26; *p* = 0.249
SPT (min)	476.5 ± 73.5	463.0 ± 64.9	489.7 ± 44.4	437.2 ± 98.5	458.8 ± 50.8	401.2 ± 69.9	462.0 ± 84.0	438.7 ± 84.9	458.0 ± 83.5	484.7 ± 41.5	456.4 ± 83.7	483.7 ± 81.7	459.2 ± 76.6	*F* (11,176) = 1.97; *p* = 0.034
Sleep Latency (min)	30.8 ± 19.1	17.8 ± 8.8	26.3 ± 17.1	27.0 ± 21.5	24.2 ± 18.3	15.9 ± 10.1	25.9 ± 22.6	18.2 ± 10.1	26.7 ± 16.8	19.8 ± 21.1	16.3 ± 13.4	32.7 ± 33.4	23.8 ± 19.8	*F* (11,176) = 1.58; *p* = 0.109
Sleep Efficiency (%)	90.8 ± 6.2	88.8 ± 7.6	86.8 ± 13.4	86.1 ± 12.4	89.9 ± 8.9	92.3 ± 5.4	89.5 ± 9.9	91.2 ± 6.3	89.0 ± 8.7	84.8 ± 10.2	90.2 ± 6.2	89.5 ± 8.4	89.2 ± 8.8	*F* (11,176) = 1.03; *p* = 0.423
REM (%)*	**23.6 ± 4.0**	**19.5 ± 3.8**	**19.9 ± 5.1**	**18.3 ± 4.2**	**22.7 ± 4.7**	**19.1 ± 5.1**	**22.1 ± 3.6**	**19.6 ± 5.7**	**24.2 ± 7.2**	**21.9 ± 4.9**	**20.7 ± 5.4**	**22.7 ± 6.5**	**21.2 ± 5.3**	***F* (11,176) = 2.31; *p* = 0.011**
REM (min)*	**100.1 ± 15.0**	**79.6 ± 18.2**	**86.4 ± 32.2**	**70.5 ± 31.0**	**94.0 ± 26.1**	**71.6 ± 25.9**	**90.4 ± 22.0**	**81.2 ± 34.4**	**100.5 ± 36.8**	**90.8 ± 26.9**	**85.1 ± 24.3**	**100.2 ± 38.7**	**87.8 ± 29.2**	***F* (11,176) = 2.39; *p* = 0.009**
REM Latency (min)*	**57.2 ± 20.4**	**60.6 ± 23.6**	**74.4 ± 30.0**	**71.1 ± 29.0**	**79.3 ± 16.2**	**68.8 ± 22.2**	**69.2 ± 24.8**	**69.6 ± 17.6**	**54.8 ± 15.6**	**64.3 ± 20.4**	**83.5 ± 22.0**	**64.1 ± 22.1**	**68.0 ± 23.3**	***F* (11,176) = 2.34; *p* = 0.010**
REM Stability (min)	7.4 ± 3.6	7.9 ± 4.3	6.9 ± 2.3	6.8 ± 2.5	5.6 ± 2.5	6.0 ± 3.4	5.3 ± 2.9	6.5 ± 4.6	4.8 ± 3.1	5.4 ± 1.9	5.9 ± 2.5	6.1 ± 3.5	6.2 ± 3.2	*F* (11,176) = 1.28; *p* = 0.241
SWS (%)*	**16.4 ± 7.1**	**18.0 ± 8.4**	**16.6 ± 10.8**	**15.6 ± 9.5**	**17.7 ± 5.2**	**17.1 ± 7.5**	**15.0 ± 8.1**	**15.3 ± 8.8**	**6.5 ± 6.4**	**10.5 ± 9.1**	**13.0 ± 8.9**	**15.1 ± 9.9**	**14.8 ± 8.7**	***F* (11,176) = 2.19; *p* = 0.017**
SWS (min)	69.8 ± 31.0	72.4 ± 34.0	73.0 ± 55.6	58.8 ± 37.4	72.4 ± 22.4	63.4 ± 30.2	63.6 ± 40.8	60.2 ± 32.9	29.6 ± 31.8	43.6 ± 39.5	51.8 ± 32.0	64.3 ± 39.8	60.6 ± 37.2	*F* (11,176) = 1.84; *p* = 0.051
N2 (%)	49.2 ± 7.4	51.4 ± 6.2	53.2 ± 9.2	51.1 ± 7.2	50.8 ± 5.1	52.2 ± 6.2	52.7 ± 6.5	54.5 ± 7.3	53.9 ± 9.7	56.0 ± 7.5	58.2 ± 8.1	53.0 ± 9.6	53.0 ± 7.8	*F* (11,176) = 1.81; *p* = 0.055
N2 (min)	215.2 ± 55.6	210.7 ± 39.0	224.1 ± 48.3	193.7 ± 61.4	209.4 ± 36.6	191.7 ± 32.0	216.2 ± 45.3	216.7 ± 46.9	217.9 ± 53.1	228.0 ± 34.1	238.4 ± 48.5	225.6 ± 43.1	215.9 ± 46.7	*F* (11,176) = 1.50; *p* = 0.136
Arousal Index (n/h)	15.8 ± 9.8	11.3 ± 5.6	14.0 ± 6.2	21.9 ± 14.6	17.5 ± 7.1	20.5 ± 14.9	14.7 ± 7.7	18.0 ± 7.6	19.5 ± 9.0	19.4 ± 8.2	15.7 ± 7.8	16.4 ± 9.7	17.0 ± 9.8	*F* (11,176) = 1.52; *p* = 0.128
Awakening Index (n/h)	2.6 ± 1.0	2.7 ± 1.0	2.5 ± 0.7	2.8 ± 1.2	2.1 ± 1.0	2.6 ± 1.1	2.1 ± 0.8	2.7 ± 1.3	2.8 ± 0.8	2.7 ± 0.9	2.3 ± 0.7	2.2 ± 0.8	2.5 ± 1.0	*F* (11,176) = 1.19; *p* = 0.295
PLM Index (n/h)	28.8 ± 27.8	49.4 ± 43.1	17.1 ± 7.2	8.6 ± 6.4	11.0 ± NA	64.7 ± 59.0	30.4 ± 42.5	9.1 ± NA	14.5 ± 13.4	24.1 ± 12.3	NA	35.0 ± 28.1	27.2 ± 29.0	*F* (10,28) = 1.36; *p* = 0.250
**Number of Patients (PLM Index ≥ 5)**	**20 (6)**	**13 (4)**	**13 (5)**	**15 (4)**	**12 (1)**	**18 (2)**	**19 (5)**	**13 (1)**	**12 (4)**	**15 (3)**	**18 (0)**	**20 (4)**	**188 (39)**	

Given are average standard deviation and the number of patients for each month and the year as a whole.

NA = not available; SD = standard deviation; TST = total sleep time; SPT = sleep period time; REM = rapid eye movement; SWS = slow wave sleep; N2 = non-REM sleep stage 2; (min) = sleep stage duration in minutes; (%) sleep stage as a percentage of TST; sleep efficiency = TST/ SPT; REM stability = REM to not-REM transitions/ REM (min); PLM = periodic limb movement (only included patients with a PLM Index ≥ 5).

*Significant after Bonferroni-Holm correction for multiple comparison applied to each of the 5 sleep parameter groups; analysis of variance for linear mixed-effects model (sleep parameter ∼ month).

Post hoc: multiple comparison with corrections for false discovery rate were significant for: (light gray background = months with significantly lower values; dark gray = significantly higher values); REM (%) higher in January than April [*t* (33) = 3.80; *p* = 0.001; *d* = 1.74 (large effect size)]; REM (min) higher in January than February [*t* (31) = 3.52; *p* = 0.001; *d* = 1.74], April [*t* (33) = 3.73; *p* = 0.001; *d* = 1.68] and June [*t* (36) = 4.20; *p* > 0.001; *d* = 1.75]; REM latency (min) higher in November than January [*t* (36) = 3.83; *p* > 0.001; *d* = 0.70 (medium effect size)] and September [*t* (28) = 3.89; *p* = 0.001; *d* = 0.74]; higher in May than September [*t* (22) = 3.76; *p* = 0.001; *d* = 1.79]; SWS (%) lower in September (median = 6.14) than January (median = 15.67; *U* = 35.00; *p* = 0.001), May (median = 19.42; *U* = 13.00; *p* = 0.001), and June (median = 18.59; *U* = 30.00; *p* = 0.001). Bold values represent the sleep parameters with a significant LMM and the number of patients for each month.

### 2.3. Statistical analysis

Analysis of variance for linear mixed-effects model (LMM) was performed in MATLAB (The Mathworks, Inc., Natick, MA, USA) using sleep parameters as dependent factors and “month of record” as the fixed factor. The covariates of gender (self-declared female vs. self-declared male), age group (≥ 60 vs. < 60 years), and diagnosis (see Table 1) were examined. These factors were removed from the LMM since the covariates were not consistently significant across the sleep parameters and did not change the significance of the results. Only sleep-related breathing disorders (srBD) showed a significant covariance to most REM sleep parameters, but including srBD in the model did not change the results.

Bonferroni-Holm (Groppe, 2022) was used for multiple comparison correction between the LMMs. Shapiro-Wilk test (BenSaïda, 2022) determined the use of either a two samples *t*-test or a Mann-Whitney-Wilcoxon test (Cardillo, 2009) for *post hoc* analysis. To account for the number of comparisons between months, the less conservative False Discovery Rate (Benjamini and Hochberg, 1995; Gerber, 2022) was applied. Data were summarized by extracting the means (referred to as: monthly means) and SD for each month.

Furthermore, the dataset was visualized by applying a moving average (MvA) with a 90-day window and a 7-day slide to each sleep parameter. Since the study only contains data collected during one year, the dataset was extended by repetition. For example, the window centered at 03.01.2019 is averaging the data collected between 19.11.2019–31.12.2019 and 01.01.2019–17.02.2019. A sample autocorrelation was conducted to identify the lag with the smallest autocorrelation (MinLag) to identify at what period the MvA pattern is reversed. The MvA data was also tested for autocorrelations using the Ljung-Box Test to evaluate if there is a structure within the time series.

## 3. Results

**FIGURE 1:**
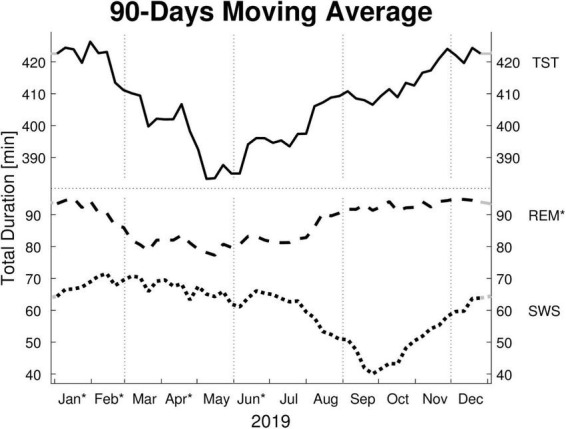
Moving averages [window: 90 days; slide: 7 days (centered on Thursdays)] for selected sleep parameters. Displayed are: total sleep time (TST), rapid eye movement sleep time (REM), and slow wave sleep time (SWS) in minutes (min). Dotted vertical lines indicate the meteorological start for each season (winter, spring, summer, and autumn). Dotted horizontal line indicates a break in the *y*-axis. Residuals of all sleep parameters are not independently distributed (Ljung-Box Test: *p* < 0.001, lag: 1–30 weeks) and therefore exhibit serial correlation. * *y*-axis: REM’s monthly means show a significant effect for “month of record” within a mixed linear model analysis of variance. * *x*-axis: The months’ *post hoc* displays a significant difference to at least one other month.

**FIGURE 2:**
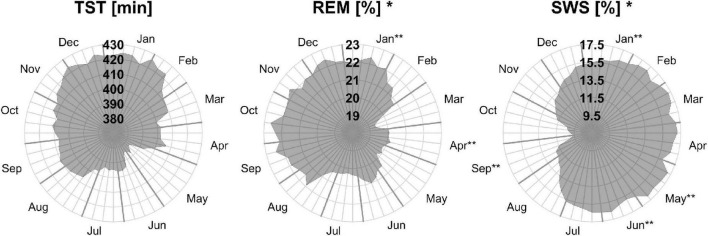
Moving averages [window: 90 days; slide: 7 days (centered on Thursdays)] for selected sleep parameters. Displayed are total sleep time in min (TST, **left**), rapid eye movement as a percentage of TST (REM, **middle**), and slow wave sleep as a percentage of TST (SWS, **right**). Gray outward-going lines represent calendar weeks, with the last Thursday of the month being thicker. *The sleep parameters’ monthly means show a significant effect for “month of record” within a mixed linear model analysis of variance. **The months’ *post hoc* displays a significant difference to at least one other month.

Monthly means of sleep parameters with SD, number of patients per month, and LMM results are presented in Table 2. Figures 1, 2 show the MvA of TST, REM sleep, and SWS. The Ljung-Box Tests indicate the existence of significant serial correlations (*p* < 0.001) for all sleep parameters.

The LMM was not significant for TST, SPT, sleep latency, or sleep efficiency. The monthly mean of the TST reaches a maximum difference between January and June of ∼62 min. Its MvA displays a clear pattern influenced by the seasons, with longer sleep in winter and shorter in spring. Between January and May, the patients’ MvA of TST decreased by 43 min. Though not displayed in Figure 1, SPT shows a similar pattern, losing 55 min of SPT between January and June. For both parameters, as well as sleep latency, the MinLag occurred at 27 weeks. This is very close to half a year (26 weeks). Sleep latency had its peak in January, but did not reach its trough till October (ΔMvA ≈ 9 min). Sleep efficiency fluctuated much stronger than the other parameters (MinLag = 14 weeks) but only by up to 3.6%.

Three of the four REM sleep parameters displayed a significant main effect of “month of record.” In September, December, and January the amount of REM (min) (*F*_11,176_ = 2.39, *p* = 0.009) is relatively high (monthly means ≈ 100 min) and in April and June low (monthly means ≈ 70 min). A similar time course is displayed by REM (%) (*F*_11,176_ = 2.31, *p* = 0.011), with its highest monthly means in December and January at ≈ 22% and lowest monthly means in April and June at ≈ 19%, see Figure 2). REM (%) and REM (min) both have a near identical MvA pattern and a MinLag of 27 weeks.

Rapid eye movement latency also showed a significant main effect of “month of record” (*F*_11,176_ = 2.34, *p* = 0.010). It peaks twice, in May and November, and has troughs in January and September. The fluctuation between the seasons can be seen in its MvA pattern with a MinLag of 14 weeks (close to the 13-week length of a quarter of a year).

No significant difference in variation was found in REM stability between months. Its MvA pattern peaks in February, dips to its lowest in September (ΔMvA = 2.25 min), and came with a late occurring MinLag (29 weeks).

Slow wave sleep (%) showed a significant main effect of “month of record” (*F*_11,176_ = 2.19, *p* = 0.017). Mostly stable throughout the year with peak values from December to May, SWS was significantly reduced in September. While SWS (min) was not significant, its MvA displays a difference of 31 min between September and February. The MinLag of SWS (min) occurred late at 29 weeks. This reflects the steep fall and rise pattern of SWS during autumn. Furthermore, SWS (%) seemingly displays an inverse relationship to REM (%) exhibiting a clear trough in September and October (see Figure 2).

Linear mixed-effects model showed no significant effect for N2 (min and %) but displayed a similar MvA pattern to TST with peaks in November and troughs in May (MvA = 231 min and 197 min; MinLag = 27 weeks). The LMMs of arousal index, awakening index, and PLM index showed no significant effects. The MvA patterns of arousals and PLM index fluctuated strongly (MinLag = 14 weeks), while the fluctuation was less pronounced for awakening index (MinLag = 16 weeks).

To examine a possible confounding effect of insomnia diagnosis on sleep duration, two independent *t*-tests were performed on TST between the astronomical seasons of spring and summer with winter. There was a significant difference between spring and winter TST (*U*_39,42_ = 638; *p* = 0.023; *d* = 0.24) with a medium effect size and 25.5 min longer TST in winter. Both seasons contained the same number of patients with insomnia (*N* = 21).

## 4. Discussion

Data from our study obtained from a heterogeneous patient group with sleep-related disorders show a significant variation of REM-minutes and -percentage of TST, with more REM-sleep during winter and less REM-sleep during summer. REM sleep latency was significantly shorter in winter and early autumn compared to late spring and late autumn. SWS shows a rather stable course over the year except for a significant decrease in autumn.

Looking at REM sleep parameters, the present finding confirms a previous observation of significant changes occurring in REM duration between seasons (Kohsaka et al., 1992; Askenasy and Goldstein, 1995). It has been demonstrated that higher-intensity light exposure suppresses subsequent REM (%) (Wams et al., 2017). Longer photoperiods, which are common in summer, are related to shorter duration of melatonin secretion (Wehr, 1991) and under natural light conditions (camping settings) were shown to be linked to delayed melatonin onset (Wright et al., 2013; Stothard et al., 2017). This results in stronger entrainment and later dim light melatonin onset, which has previously been shown to accompany decreases in REM sleep (Cajochen et al., 1997; Gordijn et al., 1999). The significant reductions in REM sleep in April and June could therefore be related to stronger circadian entrainment in summer (Roenneberg et al., 2007; Zerbini et al., 2021). In winter, entrainment effects are said to be reversed due to insufficient light exposure (Adamsson et al., 2017), which fits the observed significant increase in REM duration in January.

Since SWS is generally under homeostatic control (Borbély, 1982; Dijk et al., 1989), a longer daily wake time in summer should at most lead to deeper, and possibly longer SWS. Previously a 5.5% decrease in SWS (% of SPT) in winter compared to summer has been observed (Kohsaka et al., 1992) and SWS (% of TST) was found to accumulate higher in participants exposed to higher maximum light intensities (Wams et al., 2017). A significant decrease of SWS (% of TST) during autumn was therefore unexpected. The underlying factors that lead to these findings can only be speculated on.

This is one of few studies investigating seasonal changes in PSG-documented human sleep architecture that looks for subtle changes by comparing months and not just seasons. While the present study did not find a significant difference in objective sleep duration from the PSG-recorded data measured by month, a decrease in TST is evident from the MvA displayed in Figure 1. Monthly comparison enables one to identify more precise temporal changes. For example, in another study, there seems to be a clear increase in REM (min) between October (M ± SD ∼ 46 ± 4 min) and November (∼ 54 ± 4 min) that might be masked in their statistical analysis, since both months are pooled in their regional definition of autumn (Askenasy and Goldstein, 1995). However, the trade-off for higher precision, when using monthly comparisons instead of comparing seasons, is a reduction in statistical power. Parameters of sleep duration and sleep architecture display very large SDs, indicating a high variability between patients. On average the monthly TST SD is 71.5 min, compared to the maximum TST monthly mean difference of 61.7 min. Therefore, the lack of significant variance between months might be due to an insufficient amount of participants.

Recording patient data in a neurologic/psychiatric sleep laboratory has the advantage of allowing patients to both choose their “light off” time and to be able to “sleep in,” which can be argued to be essential for measuring sleep duration. The patients in this study slept substantially longer than in comparable studies (Askenasy and Goldstein, 1995; Herer and Lavie, 1997). One of those authors reported that the patients were awakened (Herer and Lavie, 1997), and it can be assumed that this happened in both studies. This is the norm in most sleep laboratories, especially those focused on internal medicine, where patients are generally scheduled at a set time and sent to bed once the setup is complete. On the other hand, the present study was limited by the variety of diagnosed patients with different diseases and the various intakes of different medications.

The benefits of investigating available data from a large and diverse group of patients must be weighed against collecting data from a most likely smaller group of selected healthy participants. While the latter would be a preferable scenario, the former enabled one to quickly analyze a substantially large participant pool that is mostly spaced out evenly throughout the year. The sleep architecture of insomnia is marked by prolonged sleep latency and strongly reduced TST, but the perception of reduced sleep tends to be some more severe than PSG data indicates, likely/possibly due to an increase of “micro-arousals” and alpha wave intrusion (Erman, 2001). Studies investigating the seasonality of insomnia either find no changes between seasons (Itani et al., 2016), or an increase of insomnia in winter (Husby and Lingjaerde, 1990; Friborg et al., 2012). The fact that the dataset shows a seasonal difference, as opposed to the expected changes, could indicate that these changes may be even greater if generalized to a healthy population. Nevertheless, it was important to carefully account for confounding factors in the patient group. Since certain medications, that are prevalent to be taken by a neuropsychiatric cohort, interfere with sleep in multiple ways, a large number of patients had to be excluded from the analysis. Potentially, further research could be conducted by analyzing multiple years of sleep data to replicate these findings and to increase monthly group size.

Seasonal changes in sleep architecture may have implications for recommendations regarding sleep routines. For many people, the time to wake up is more strongly controlled by their employer’s business hours or school times than by their internal clock. Adjustment of sleep schedule can, therefore, only be controlled by choosing the time to go to bed. Nevertheless, keeping to the same time is widely recommended and often enforced religiously on children. Our findings suggest that improvements can be made by accounting for the increased sleep need in winter, by going to bed earlier. Furthermore, the current twice-annual turning of the clock may facilitate adjustments to seasonal changes in human physiology underlying the findings we report here. For example, standard time in winter allows for more natural light exposure during morning commutes. On the other hand, the reduced sleep need in summer can result in longer periods of wakefulness prior to school or business hours that could be better spent during spare time in the afternoons and evenings. Especially younger people and women potentially benefit from daylight saving time this way since they were shown to exhibit stronger seasonality (Kasper et al., 1989).

In conclusion, the observed significant change in prolonged REM sleep duration in winter vs. spring was in line with previous studies. The data indicate that sleep architecture in patients with sleep disorders is affected by seasonal changes, even in an urban environment with low natural light exposure and high light pollution. We do not intend to state hard facts about the severity of changes in sleep duration between seasons in the general population but wanted to highlight that changes can be observed in a sizeable subpopulation. Since the 90s, the research on the seasonality of sleep architecture has halted. While experimental differences in sleep architecture in response to changing light exposures are well documented, the effect of season on human sleep is thought to be negated by our unlimited access to light and temperature sources, especially in an urban environment. Previously we discovered significant changes between seasons in the subjectively reported sleep duration of a homogenous group (Kunz et al., 2021). In our retrospective study, we were able to obtain an unusually large dataset for a study in this field. Being able to demonstrate these differences objectively in a heterogeneous patient group is of value for sleep schedule recommendations to patients and can hopefully be used as an incentive to justify larger studies on this topic again. Furthermore, it bears investigation if these changes can be directly related to underlying factors, such as sunshine duration and temperature changes and if the observation of a decrease in SWS in autumn can be replicated for further years. Overall, potential benefits of adjusting sleep behavior with the changing seasons seem evident, at least for individuals with insomnia.

## Data availability statement

The original contributions presented in this study are included in the article/supplementary material, further inquiries can be directed to the corresponding author.

## Ethics statement

Ethical review and approval was not required for the study on human participants in accordance with the local legislation and institutional requirements. The patients/participants provided their written informed consent to participate in this study.

## Author contributions

JZ, FB, and DK designed the experiment. AS performed the study and drafted the article. JZ, AS, and KW analyzed the data. AS and KW prepared the figures. All authors edited and reviewed the article, and approved the final version.
